# Respiratory syncytial virus: health burden, disease prevention, and treatment—recent progress and lessons learned

**DOI:** 10.1093/femsml/uqaf003

**Published:** 2025-02-10

**Authors:** Harald Brüssow

**Affiliations:** Laboratory of Gene Technology, Department of Biosystems, KU Leuven, 3001 Leuven, Belgium

**Keywords:** respiratory syncytial virus, virology, disease burden, passive immunity, vaccinology, antivirals

## Abstract

Respiratory syncytial virus (RSV), a negative-sense single-stranded RNA virus of the Pneumoviridae family, represents the most important pathogen of lower respiratory tract infections in young infants causing yearly epidemics. RSV is also an important respiratory viral pathogen for older subjects, which is second only to seasonal influenza virus infections. RSV represents a substantial public health burden with respect to morbidity and mortality, particularly in developing countries. Prevention and treatment options would therefore lessen the global disease burden. A formalin-inactivated RSV vaccine in the 1960s induced an enhanced disease upon exposure to natural RSV. After this tragical vaccine failure, it took nearly five decades of intensive research before prevention tools were approved by health authorities. The lead was taken by passive immunity approaches with injected monoclonal antibodies directed against the fusion protein F of RSV. The elucidation of the three-dimensional structure of the F protein revealed pre- and postfusion conformations. Subsequently, structure-based antigen engineering of the F protein paved the way for development of a prophylactic vaccine. In 2023, RSV vaccines were approved for maternal vaccination to protect young infants by placental transfer of antibodies and for vaccination in older subjects. Antiviral drugs that target the RSV fusion process, the RSV replicase, or the cytoplasmic viral factories are in development. Important research papers leading to these developments are reviewed here.

## Teaser

Respiratory syncytial virus (RSV), as an endemic virus infection, does not itself represent a pandemic threat. Since respiratory viruses have caused several pandemics, claiming millions of lives, the lessons learned from RSV research might contribute to pandemic preparedness.

## Growing concerns with respiratory viruses

**Figure 1.**
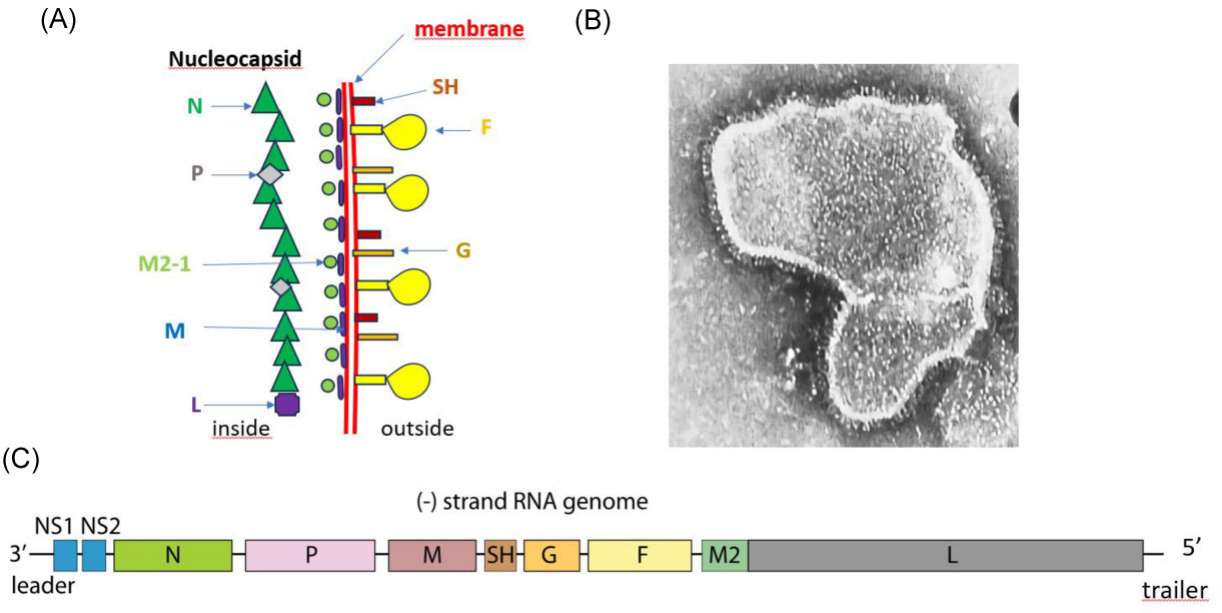
The RSV. (A) Schematic cut through viral membrane. At the left side, inside the virus, the nucleocapsid/polymerase proteins N (nucleocapsid), P (phosphoprotein), L (large polymerase), and M2-1 (transcription processivity factor). Underlying the lipid membrane is the matrix M protein. Outside of the bilayer lipid membrane, the fusion glycoprotein F, glycoprotein G, and the small hydrophobic protein SH (schematic drawing adapted from Buchholz et al. 2023). (B) Electron microscopic picture of an RSV particle (Centers for Disease Control and Prevention). (C). Genome map of RSV with 3′ leader and 5′ trailer RNA sequences and gene location for nonstructural proteins NS1 and NS2 and virus proteins N, P, M, SH, G, F, M2 (M2-1 and M2-2), and L (Swiss Institute of Biotechnology).

When looking back to pandemics claiming millions of human lives over the last century, it is striking how many were caused by respiratory viruses (list of epidemics and pandemics—Wikipedia). One is therefore well advised to keep an open eye on influenza A viruses (IAV) and coronaviruses in all efforts of pandemic preparedness. The World Health Organization (WHO) has also listed Nipah virus, a paramyxovirus, as a pandemic virus candidate. Respiratory syncytial virus (RSV) belongs to Pneumoviridae, a sister family of Paramyxoviridae (Fig. 1). RSV causes infections in humans manifesting as regular annual winter epidemics. As an endemic virus, RSV is an unlikely candidate for a future pandemic. However, the viral world is currently very dynamic. RSV cases nearly disappeared in the winter of 2020–21, only to return vigorously in 2022 with an unusual epidemiology (Abu-Raya et al. 2023, Cong et al. 2024). In 2022, newspapers started to report about a “tripledemic” where case numbers for COVID-19, influenza, and RSV disease surged and started to overwhelm hospitals. Concomitant respiratory epidemics raise concerns for dual viral infections, which might cause more severe symptoms (Franz et al. 2010). In addition, dual infections with IAV and RSV can lead—at least in cultured cells—to hybrid viral particles displaying surface glycoproteins and genomes from both viruses. Furthermore, the hybrid virus could use the RSV fusion (F) glycoprotein to infect cells lacking receptors for IAV, thus potentially extending the tropism of IAV (Haney et al. 2022). The clinical relevance of these cell culture data is however not clear. When analyzing diagnostic data from viral respiratory infections in children from Scotland, a quarter were coinfections, involving rhinovirus, adenovirus, RSV, and coronavirus, while IAV was rarely detected in association with RSV (Nickbakhsh et al. 2016). Theoretically, the interaction between two viruses could be disease enhancing or reducing (positive or negative interference). In animal studies, interaction of RSV with IAV or with human metapneumovirus was characterized by negative interference via induction of innate immune mechanisms (Piret and Boivin 2022). In clinical studies negative interference was described for RSV and rhinovirus coinfection both for viral replication and disease severity (Achten et al. 2017).

In nature, RSV circulates only in humans. Ruminants are infected by genetically distinct animal RSVs (Buchholz et al. 2023). However, closely related human RSV genome sequences were reported in sick Malayan pangolins that also carried severe acute respiratory syndrome coronavirus 2 (SARS-CoV-2)-like virus sequences (Ye et al. 2022). Disturbingly, these animals developed symptomatic disease from which they died, but they might represent rare cases of reverse zoonosis. Independent from these theoretical concerns, RSV is a major human pathogen in the very young and old subjects, representing a major global health burden.

## Epidemiology of RSV infections in infants and older adults

### Pediatric RSV

A few numbers illustrate the importance of RSV disease. Ten years ago, 20% of an annual birth cohort in the United States required outpatient medical attention during the first year of life because of RSV illness. Between 2% and 3% of all children younger than 12 months were hospitalized with a diagnosis of bronchiolitis where RSV was the etiological agent in 50% to 80% of the cases (the remainder being rhinovirus, parainfluenza virus, metapneumovirus, coronavirus, and adenovirus infections). RSV replicates first in the nasal turbinate, then propagates to the bronchioles where increased mucus production, impaired ciliary action, and sloughed epithelial cells obstruct the lumen resulting distally in trapped air that after absorption causes collapse of the alveoli (Meissner 2016). Children infected with RSV displayed fever, cough, runny nose, nasal congestion, decreased appetite, rapid and shallow breathing, nasal flaring and grunting, difficulty breathing, and apnea. Hospital admission mostly for bronchiolitis was associated with younger age (<6 months) and a higher viral load (Haddadin et al. 2021, Toepfer et al. 2024). Gastrointestinal symptoms (vomiting, diarrhea, and feeding difficulties) were widespread in RSV patients (Colosia et al. 2023). No available treatment shortens the course of bronchiolitis, and therapy is supportive and consists of fluid hydration and respiratory support (Mazur et al. 2024). Treatment with antibiotics is mostly unnecessary since <10% of RSV cases represent bacterial coinfections. Antiviral therapy has so far not shown much promise since it mostly started days after disease onset. Inhaled nebulized hypertonic salt somewhat accelerated the amelioration of the clinical score (Luo et al. 2011) while a combination of three nebulized single-chain antibodies (nanobodies) directed against the fusion protein (F) of RSV showed no clinical response despite a faster drop in viral load (Cunningham et al. 2021). Prevention is therefore considered to be more promising than treatment of RSV disease.

In the United States, yearly outbreaks of RSV infections occur between November and April; 2.1 million children suffer from RSV infections, which require medical attention, 60 000 of them were hospitalized (Hall et al. 2009). The annual rate of RSV-associated hospitalization was 17 per 1000 subjects. Bronchiolitis was the main diagnosis in hospitalized infants, while pneumonia and asthma dominated in older children. Labored respiration requiring supplemental oxygen was observed in nearly all hospitalized children. Risk factors for hospitalization were a previous episode of wheezing and prematurity.

Birth cohort studies from South Africa showed that one in five lower respiratory tract infections (LRTIs) were associated with RSV, a third required hospitalization (Zar et al. 2020). Risk factors for RSV-associated LRTI were maternal smoking, maternal human immunodeficiency virus infection, season of birth, and young child age. In a European birth cohort study, 1.6% of the children were hospitalized with RSV acute respiratory infection (ARI). The rate was two-fold higher in Spain than in Finland, highest in infants 1 to 3 months of age, and higher in infants born in autumn. Median duration of RSV ARI hospitalization was 3 days. An equal proportion of subgroup A and B RSV infections was described (Wildenbeest et al. 2023).

For 2019, a global burden of 33 million RSV LRTI episodes was estimated in children younger than 5 years; 95% occurred in low- and middle-income countries (LMICs) where up to 80 episodes per 1000 children were observed (Li et al. 2022). About 5.5 million episodes showed chest wall indrawing and 3.6 million episodes led to hospital admissions; infants younger than 6 months represented nearly half of the hospitalizations. A quarter of the hospitalized children showed hypoxemia at admission, indicative of severe disease. Inhospital case fatality rates (CFRs) were 0.1% and 1.4% in high and low-income countries, respectively. When estimating in- and out-of-hospital deaths together, one in 50 childhood deaths were attributed to RSV disease (Li et al. 2022). Hospitalization rates for RSV increased between 2015 (Shi et al. 2017) and 2019, but inhospital death rates decreased over time possibly reflecting ameliorated treatment options. Other groups confirmed a global CFR decrease from 2% to 1% between 1990 and 2016, and attributed it to the global decrease of poverty (GBD 2016 Lower Respiratory Infections Collaborators 2018). After *Streptococcus pneumoniae*, RSV was the second leading etiology of LRTI mortality. Globally, childhood wasting was the greatest risk factor for LRTI mortality, followed by indoor air pollution and missed antibiotic use during an LRTI episode.

Preterm children display a less mature immune system, smaller airways, and diminished maternal antibody transfer than term infants, rendering them more vulnerable to severe RSV disease. Premature infants represented 25% of pediatric RSV hospitalization, but corresponded to 40% of RSV-associated mortality in children globally (Wang et al. 2024). Early preterm infants showed a two-fold higher hospitalization rate than term infants. Also, congenital heart disease, tracheostomy, and chronic lung disease were associated with a two-fold higher risk for severe RSV disease (intensive care, longer hospital stay). Living with two or more young children in a household was a further strong risk factor for RSV hospitalization.

### Adult RSV

Overall, symptoms in adult RSV patients were more severe than in adult IAV patients (Colosia et al. 2023). In China, older adult RSV patients showed longer hospitalization stays than in the United States, 40% displayed pneumonia and 22% bronchitis, and most patients had underlying medical conditions. Supplementary oxygen and mechanical ventilation were needed in 68% and 11% of older Chinese patients, respectively, and 9% died within a month (Lee et al. 2013).

In the United States, RSV infection developed annually in 3% of healthy older adults and in 10% with cardiovascular comorbidity. RSV infections were twice as frequent as IAV infections (Falsey et al. 2005). Among elderly US subjects, RSV infection accounted for 180 000 hospital admissions per year and 14 000 annual deaths. Based on death certificates, other researchers associated an annual excess mortality of 23 000 with RSV and 27 000 with IAV infection, the majority occurring in elderly people (Hansen et al. 2022). Based on reverse transcription-polymerase chain reaction (RT-PCR) tests in nasopharyngeal samples and serology results, 1.4 million outpatient visits, 160 000 hospitalizations, and 10 000 deaths for RSV infections were estimated annually among older US citizens (McLaughlin et al. 2022). The annual RSV-associated hospitalization rate was with 42 000 in 50- to 64-year-old subjects still substantial and was associated with obesity and diabetes as risk factors.

Savic et al. (2023) conducted a meta-analysis for RSV infections in adults older than 60 years from high-income countries (United States, Europe, and Japan). Attack rate for RSV-associated disease in this age group was 1.6%, and hospitalization rate was 0.15%, which showed an inhospital CFR of 7%. For 2019, these authors estimated 5 million cases, half a million hospitalizations, and 33 000 deaths among older adults in these three high-income regions. Data on RSV disease burden in older people from developing countries are scarce, but probably substantial since rates of undifferentiated LRTI in elderly subjects were worldwide highest in South Asia (GBD 2019 LRI Collaborators 2022).

## Impact of the COVID-19 pandemic on RSV epidemiology

### Factors affecting RSV epidemiology

A typical RSV season occurs annually from late fall through early spring, with a peak during winter. A new RSV epidemic is reintroduced into the community (“epidemic seeding”) every year. Between the epidemic seasons low levels of RSV RNA were detected in wastewater samples. The persistence of the virus might therefore be assured by low-level infections in the populations between epidemic seasons (Allen et al. 2024). The yearly epidemics show sometimes a directed geographical spread (e.g. from Southeast and to the North and West of the United States) (Zheng et al. 2021). RSV is primarily transmitted through respiratory droplets (coughs and sneezes) and via contact through contaminated surfaces. Demographic factors, such as the population age distribution, susceptibility of the population and contact rates, household size, and population density, affect the shape of the RSV epidemic curve. However, epidemic peak sizes change little, less than two-fold between the years (Zheng et al. 2021). When going to viral strain level, more variability was documented: the relative contribution of subtype A and B RSV to the epidemics can vary markedly from year to year (Toepfer et al. 2024).

### Changed RSV epidemiology after 2020

During the COVID-19 pandemic, the epidemiology of RSV changed dramatically and worldwide. Practically no cases of RSV were for example observed in England during winter 2020–21 (Bardsley et al. 2023). RSV test positivity, which regularly rose to 40%–50% of viral diagnostics during the winter peaks, remained below 2%, and medical contacts for acute bronchitis or bronchiolitis were negligeable. A second anomality occurred in the second half of 2021: a marked, broad summer peak of RSV cases was observed. A third anomaly was the shift of laboratory-confirmed RSV infections in 2021 to children older than 1 year. Also in the United States, the usual December/January peak of an RSV epidemic was totally suppressed in 2020, followed by a greater than usual early October peak in 2021, and an even greater surge in October 2022 resulting in a 3.5-fold increase in emergency department visits (Winthrop et al. 2024). In the southern hemisphere, the usual mid-year RSV epidemic in the austral winter was suppressed and followed by a sharp and greater than usual peak in the austral summer together with a shift to older children (Foley et al. 2021). When comparing data from 19 countries a more complex picture emerged (Cong et al. 2024). For high-income countries, a marked drop in RSV-associated hospitalizations was observed in spring 2021 compared to spring 2020, followed by a resurge of RSV hospitalizations to prepandemic levels in spring 2022. Middle-income countries already showed an earlier drop in RSV hospitalization in mid-2020 followed by a less accentuated return of RSV hospitalizations.

### Changes in viral virulence?

A controversial point was whether the resurge was caused by more virulent RSV. In the United States, the number of high-flow nasal cannula respiratory support doubled in 2022–23, challenging the capacities of pediatric hospitals (Winthrop et al. 2024). However, the proportion of patients needing respiratory support decreased, arguing against the appearance of RSV strains with higher virulence. The most notable feature of postpandemic RSV was the higher proportion of pediatric patients older than 1 year lacking comorbidities. Also, the inhospital CFR of RSV cases tended to be lower, refuting the hypothesis that a delayed exposure to RSV leads to more severe disease (Cong et al. 2024). Likewise, data from Denmark reporting a doubling of mechanical ventilation in children hospitalized with RSV LRTI in 2021 (Nygaard et al. 2023) should be interpreted with regard to the increased case numbers. In fact, the rate of 14 complications per 1000 RSV hospitalizations remained the same. Furthermore, data from Northern Ireland showed a close temporal correlation between RSV concentrations in wastewater and the buildup of clinical cases. The viral RNA load in wastewater was much higher in 2022 than in 2021 without that the RSV case number increased in parallel, suggesting that the virulence of RSV had not increased (Allen et al. 2024).

Was the marked postpandemic RSV surge caused by viruses with higher transmissibility? Also this seems not to be the case: at least 10 different RSV lineages were detected during the postpandemic surge by whole viral genome sequencing (Adams et al. 2023). All had already circulated in the prepandemic era. With a substitution rate of 10 bases per year for its 15-kb single-stranded negative-sense RNA genome, RSV is far away from the accelerated evolution seen in SARS-CoV-2 genomes.

### Lockdown and reduced viral exposure

The most likely explanation for both the suppression of the RSV epidemic in the winter 2020–21 in the northern hemisphere (and in mid-2020 of Australia) and the subsequent surge of RSV cases with a shift to older children can be found in the lockdown measures imposed by governments for the control of the COVID-19 pandemic before vaccines became available (UK: March 2020, November 2020, and January 2021). Lockdowns reduced the mobility of the population and the contact rate between people, which should have an immediate effect on a viral respiratory infection. The dramatic drop in RSV case numbers during the application of nonpharmaceutical interventions (NPIs) against COVID-19 suggests a strong temporal association between these two events. The resurge of RSV infections after relieving NPI measures indicates a causal connection. The lockdowns also provide a plausible explanation for the shift of RSV hospitalizations to older children. Lack of viral exposure during NPI restrictions caused waning humoral immunity (“immune debt”; Billard and Bont 2023) in the entire population, but particularly in young infants (Reicherz et al. 2022, den Hartog et al. 2023) creating an increased pool of immunologically RSV-naïve infants, including older children. The cellular immune response against RSV showed no waning in adults over the same period (Reicherz et al. 2022), which might explain why the post-NPI surge was particularly prominent in the pediatric population. The changed RSV epidemiology during and after the COVID-19 pandemic can thus easily be rationalized as lockdown effects. Studies on less onerous NPI such as face mask use and hand washing on RSV infections are scarce and not conclusive (Dallagiacoma et al. 2024). The application of lockdowns for containing the COVID-19 pandemic thus also represents a worldwide natural experiment providing revealing insights into the epidemiology of viral respiratory infections (Messacar et al. 2022). Apparently, the resumed social contacts after relieving lockdowns and social restrictions had a stronger impact on RSV infection chains than meteorological factors (cold and humidity) as demonstrated by the off-season RSV endemic peak.

## Enhanced disease after vaccination with formalin-inactivated RSV

### Failed vaccine trials

In the late 1960s, a formalin-inactivated RSV (FI-RSV) vaccine was tested in US children. Low-level neutralizing antibodies (Nab) were induced in vaccinees, but failed to offer clinical protection. In fact, the FI-RSV vaccine induced an exaggerated clinical response following natural exposure to RSV. In one trial, 69% of the vaccinees compared to 9% of the controls developed pneumonia (Kapikian et al. 1969). In another trial, 80% of FI-RSV vaccinees required hospitalization compared to 5% of control children receiving a parainfluenza vaccine (Kim et al. 1969). Clinical reports from two male toddlers who died from enhanced RSV disease (ERD) demonstrated prolonged cough, tachypnea, high fever, and respiratory failure (Polack et al. 2021). Autopsy material revealed bronchiolar inflammation with elevated levels of neutrophils and eosinophils. A transcriptome analysis demonstrated a suppression of the type I interferon response. A colocalization of Immunoglobulin G (IgG) and complement was detected in the bronchioles of the two toddlers causing tissue injury (Polack et al. 2002).

### Immunological causes for the failure

Several studies have explored the potential mechanisms underlying the formalin-treated vaccine’s failure. Formalin treatment for example leads to an abundance of carbonyl groups in the RSV vaccine preparation, which favors in BALB/c mice a Th2-biased immune response inducing lung eosinophilia (Moghaddam et al. 2006). Chemical modification of the viral proteins by the inactivating agent might be at the basis of the vaccine’s problems. Replication-competent RSV, but not FI-RSV, induced a CD8 T-cell response against epitopes on the M2 and F viral proteins in mice. However, when FI-RSV-immunized mice were boosted with a vaccinia vector expressing the M2 protein, the animals showed upon RSV challenge a reduced pulmonary eosinophilia. Apparently, RSV M2-specific CD8 T cells reduced the Th2-mediated pulmonary pathology (Olson and Varga 2007). The limited range of cellular immune responses to viral antigens in the formalin-treated vaccine might add to the problems with that vaccine.

A study by Delgano and colleagues showed that, in BALB/c mice, FI-RSV and ultraviolet radiation (UV) -inactivated RSV induced low-avidity IgG antibodies with low neutralization activity. Lack of antibody affinity maturation was the result of poor Toll-like receptor (TLR) stimulation by these inactivated viruses, demonstrating a possible mechanism responsible for the lack of protection by the antibody response in this model. Supplementation of UV-RSV vaccine with the TLR agonist lipopolysaccharide promoted antibody avidity maturation, stimulated Nab titers, and protected against ERD (Delgano et al. 2009). These results suggest that the inactivated RSV vaccine may be rendered safe by stimulating affinity maturation by TLR agonists.

RSV replicating intracellularly in cell culture experiments interacts with numerous host factors: F protein is recognized by TLR4, single-stranded RSV RNA by TLR7, and double-stranded RSV RNA by still other factors leading to transcription factor activation and antibody affinity maturation. Since these activation processes do not occur with inactivated RSV vaccines, cellular responses differ fundamentally between live and inactivated RSV vaccines explaining further aspects of the FI-RSV vaccine’s failure (Varga 2009). Vaccine antigens not processed in the cytoplasm may therefore present a risk in RSV-naïve children, which blocked the development of pediatric RSV vaccines (Acosta et al. 2015). Since children with prior exposure to RSV did not develop ERD upon vaccination with FI-RSV, ERD is unlikely in adults since RSV-specific antibodies are nearly universally acquired early in childhood (Brüssow et al. 1991). Not surprisingly, the development of passive immunity to RSV with monoclonal antibodies and maternal vaccination took advance over the development of RSV vaccines for children.

## Passive immunity with monoclonal antibodies

### Palivizumab

Following the tragic outcomes with the early FI-RSV vaccines, pediatricians had for a long time nothing to offer to the parents of children at high risk of developing severe RSV disease. The situation changed in 1998 when the US Food and Drug Administration (FDA) approved palivizumab (Synagis, AstraZeneca), a humanized monoclonal antibody (mab) directed against the F protein of RSV. Phase 3 clinical trials demonstrated that intramuscular injections of palivizumab reduced the rate of RSV-related hospitalizations in premature infants (1.8% vs 8.1% in placebo) and in infants with bronchopulmonary dysplasia and congenital heart disease (Gonzales et al. 2024). In its latest recommendation, the American Academy of Pediatrics, however, did not back the use of palivizumab in otherwise healthy preterm infants (Caserta et al. 2023). Another problem with palivizumab was the cost incurred for five monthly injections to protect a child over one RSV season.

### Nirsevimab development

Therefore, biotechnologists tried to address the problem by developing a mab that protects at lower concentrations and displays longer half-lives after injection, thus reducing cost by using a single intramuscular injection (Zhu et al. 2017). The researchers selected IgG1 mabs from memory B cells of human donors, engineered the mab-binding site by mutagenesis to increase RSV affinity. They also modified the constant part of the mab to extend its serum half-life by introducing a triple amino acid (aa) substitution that increased the antibody’s affinity for the human FcRn receptor, allowing for recirculation of the mab. This resulted in humans in an up to four-fold increase in half-life compared with the parental antibody and a decreased immune response against the mab. After screening many mabs in microneutralization tests, the researchers selected one mab that was 150-fold more potent than palivizumab. This mab, called nirsevimab, binds a highly conserved antigenic site, namely epitope ф on the top of the prefusion conformation of the F protein, conferring broad neutralization of both RSV A and RSV B subtypes. Palivizumab binds antigenic site II located at the lateral side of the prefusion F protein. Nirsevimab demonstrated a 9-fold higher potency than palivizumab in reducing lung and nose RSV titers in cotton rats. The protective concentration of the mab was maintained in the serum of monkeys after a single intramuscular injection (half-life of 70 days), paving the way to clinical trials.

### Nirsevimab clinical trials

In healthy preterm infants, nirsevimab application reduced medically attended RSV LRTI (2.6% vs 9.5% in placebo recipients) and RSV-specific hospitalizations (0.8% vs 4.1%) (Griffin et al. 2020). No safety concerns were raised and 90% of the subjects still had protective serum mab concentrations after 5 months. Only 5% of the treated infants developed anti-mab antibodies. A second trial using nirsevimab plus placebo or nirsevimab plus palivizumab in preterm and at-risk infants reproduced the protective effect of nirsevimab, without demonstrating a synergistic effect of giving in addition to palivizumab (Domachowske et al. 2022). The MELODY trial enrolled mostly healthy term infants (not eligible for palivizumab). Nirsevimab prevented 70% of RSV events compared to placebo (medically attended RSV: 1.2% vs 5%; RSV hospitalizations: 0.6% vs 1.6%) (Hammitt et al. 2022). Serum nirsevimab concentrations decreased in children with a half-life of 70 days. Adverse events (AEs) were observed with similar frequency in the treatment and placebo groups. The researchers calculated that 15 RSV hospitalizations were averted for 1000 children treated with nirsevimab. Based on these data, nirsevimab under the trade name Beyfortus was approved for Europe in 2022 and in 2023 for the United States with the indication of prevention of RSV LRTI in neonates and infants during their first RSV season. An efficacy of 77% was calculated for nirsevimab with follow-up data for medically attended, hospitalized and very severe RSV LRTI (Muller et al. 2023). The pragmatic European HARMONIE trial enrolled healthy infants and run into the resurge of the RSV epidemic in the winter 2022–23 (Drysdale et al. 2023). Nirsevimab reduced RSV hospitalization (0.3% vs 1.5% in placebo recipients) suggesting an 83% prevention efficacy. Very severe RSV infection defined by the need for supplemental oxygen was also reduced (0.1 vs 0.5%). AEs were observed with similar frequency in the treatment and control groups.

A pooled analysis of randomized controlled trials concluded that nirsevimab has the potential to change the landscape of infant RSV disease by reducing a major cause of infant morbidity and the consequent burden on caregivers, clinicians, and healthcare providers (Simoes et al. 2023). It remains to be established whether the prevention of RSV infections has beneficial effects for respiratory health in children in later life, e.g. on the development of childhood asthma (Feikin et al. 2024, Zar et al. 2024).

### Real-world data

A number of real-world data on the efficacy of nirsevimab prophylaxis on reducing RSV-related hospitalization were reported for various countries (Orsi et al. 2024). In Galicia/Spain, nirsevimab was given during the winter 2023–24 RSV epidemic (Ares-Gomez et al. 2024). Overall, 0.3% of 9400 infants who received nirsevimab and 1.9% of 850 infants who did not receive nirsevimab were hospitalized for RSV-related disease, indicating an immunization efficacy of 82%. Against severe RSV disease requiring oxygen support the efficacy was 87%. The authors calculated that 25 infants needed to be immunized to avoid one RSV hospitalization for an LRTI.

In France, 230 000 single injection doses of nirsevimab had been applied to newborns and young infants free of charge during the 2023–24 RSV season. In a post-licensing case–control study, 690 patients hospitalized with an RSV-associated bronchiolitis were compared to 345 matched control infants visiting the emergency department for conditions other than bronchiolitis (Assad et al. 2024). Overall, 8.7% of cases and 28% of controls had received nirsevimab indicating an immunization efficacy of 83%. Against severe infection (intensive care and ventilatory support) the efficacy was at least 67%.

### Selection of escape mutants?

The widespread use of nirsevimab in France might raise concerns for the selection of nirsevimab-resistant RSV variants. An analysis of nearly 6000 RSV genome sequences collected from various regions of the world between 2015 and 2021 (Wilkins et al. 2023) showed that this risk is small since the nirsevimab-binding site remained highly conserved. Most RSV strains containing binding-site substitutions were still neutralized by nirsevimab, except for a few RSV B variants, which represented <1% of the analyzed viruses. These data demonstrate that the F protein from RSV evolves less rapidly than the neutralizing antigen of other respiratory viruses (IAV hemagglutinin, SARS-CoV-2 spike protein), which makes passive immunity against RSV with a mab a realistic task. To address the risk of selecting escape mutants by large-scale nirsevimab application, French researchers (Fourati et al. 2024) sequenced ∼500 RSV genomes from infants treated or not treated with nirsevimab during the 2023–24 season and tested them for neutralization by nirsevimab. RSV-A isolates did not reveal any substitution in the binding site of nirsevimab. However, two of 24 RSV-B breakthrough infections had resistance-associated substitutions underlining the importance of continued molecular surveillance. US clinicians consider extending nirsevimab use to immunocompromised adults (transplantation patients, NCT05921903; https://clinicaltrials.gov) and older subjects at risk of severe RSV disease. Since immunocompromised COVID-19 patients showed an increased rate of virus evolution, the risk of selecting nirsevimab-resistant mutant RSV might be greater in these patients. Developing new mabs against escape RSV mutants might be technically and regulatorily more demanding than adapting mRNA vaccines to escape SARS-CoV-2 variants.

### Cost considerations

In the United States, the price of a single dose of nirsevimab in the private health market is US ${\$}$520 (Pecenka et al. 2024). Infant RSV treatment costs in the United States are US ${\$}$700 million annually corresponding to US ${\$}$200 per birth. Product pricing varies by country. For the UK and Canada, it was calculated that nirsevimab or maternal vaccination (US cost ${\$}$300) could be cost effective (for maternal vaccination, see the corresponding section next). Since the majority of the RSV disease and mortality burden is found in children from developing countries, the question of an economic feasibility for passive immunization in LMICs is raised. Calculations showed that RSV maternal vaccination and mab is cost efficient with a price of ∼US ${\$}$30–${\$}$40 per dose in Kenya (Koltai et al. 2023) and US ${\$}$50 for Argentina (Guiñazú et al. 2024). Calculations for 72 Gavi-eligible countries revealed that mab is more effective than maternal vaccination but also three times more expensive (Li et al. 2020). At a price of US ${\$}$4 per dose, both interventions were cost saving in LMIC (Mahmud et al. 2023). Extending nirsevimab use to LMIC therefore needs a subsidized pricing supported by the vaccine alliance Gavi. For LMIC an undisclosed price discount has been agreed for maternal vaccination but not for nirsevimab, which will put the latter out of reach for LMIC (Pecenka et al. 2024).

## Structural vaccinology and F-protein engineering

**Figure 2.**
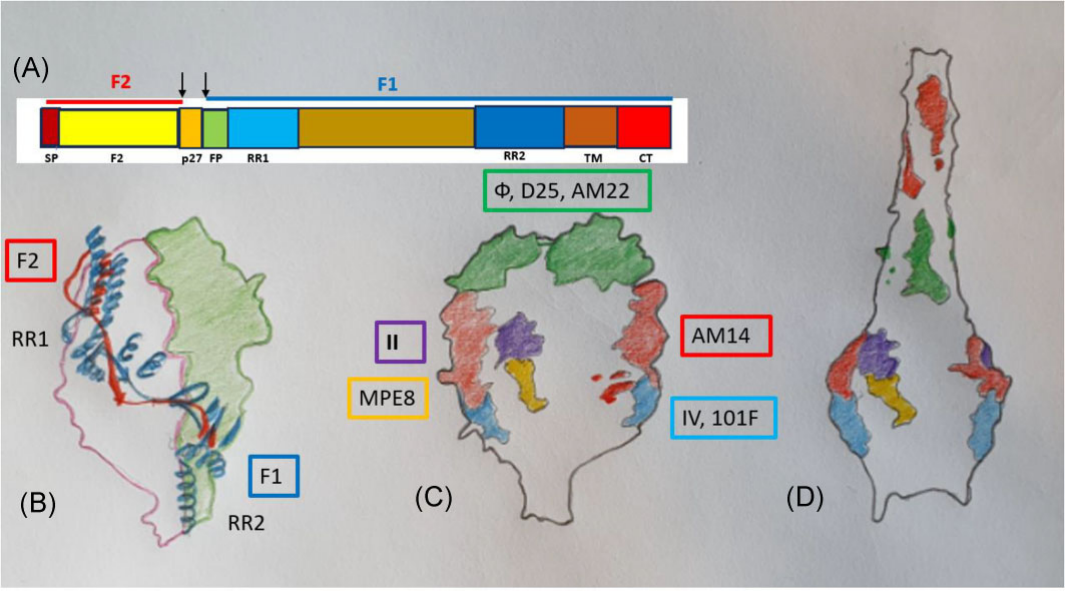
The RSV F protein. (A) Domain organization of the F0 glycoprotein; the extent of F2 and F1 proteins is indicated by horizontal lines above the protein map. SP, signal peptide; ↓ proteolytic cleavage sites; p27, protein released by cleavage; FP, fusion peptide; RR1, heptad repeat 1; RR2, heptad repeat 2; TM, transmembrane anchor; CT, cytoplasmic tail. (B) The fusion protein is a trimer consisting of three F proteins. One F monomer is shaded in green, another is outlined in pink in space-filling mode, the third F protein monomer is depicted in ribbon mode with F2 in red and F1 protein subunits in blue lining. The location of RR1 and RR2 is indicated (schematic drawing adapted from Krarup et al. 2015). Conformation and location of immunologically important epitope sites identified by distinct coloring in the prefusion (C) and postfusion (D) conformation of the F trimer. TM and CT domains are not displayed; the viral membrane is at the bottom. Site II is recognized by palivizumab (schematic drawing adapted from Che et al. 2023).

The passive immunity data with mabs demonstrated that protective antibodies can be raised. Over the last decade, structural biology data of the RSV fusion protein F, the target of potent neutralizing antibodies, likewise provided a new stimulus for RSV vaccine development (McLellan et al. 2013a). The F protein exists in two antigenically distinct forms: a metastable pear-shaped trimer prefusion conformation that spontaneously rearranges into a stable more needle-like postfusion conformation (Fig. 2). Both conformations present epitopes, which are targets for neutralizing mabs. Palivizumab recognizes antigenic site II, which is exposed at the apex of the postfusion conformation, but is located on the lateral side of the prefusion conformation. More potent mabs than palivizumab such as mab D25 were developed that recognize the prefusion-specific quaternary antigenic site ф, located at the top of the prefusion protein. National Institutes of Health (NIH) researchers used protein engineering to stabilize the prefusion conformation for optimal presentation of the antigenic site ф (McLellan et al. 2013b). To achieve this goal, they used three strategies: they added the T4-phage fibritin trimerization domain (“foldon”) to the C terminus of the F ectodomain; they introduced new cysteine pairs to prevent the rearrangement of the pre- into the postfusion conformation; and they filled caves in the prefusion conformation with hydrophobic aa substitutions. Then they screened engineered F proteins for high expression yield and stability and tested the best candidates for immunogenicity in mice and rhesus macaques. They thus selected DS-Cav1 (displaying a specific DiSulfide bond constellation and a specific Cave filling) as the best candidate. In adult human volunteers, DS-Cav1 protein F vaccine induced a 12-fold and a 4-fold increase in Nab titer against RSV A and RSV B, respectively, which was independent of dose or adjuvant addition (Crank et al. 2019). Antibodies were directed against both exclusive pre-F and shared pre-F and post-F epitopes. The pre-F-specific antibody correlated with RSV neutralization. DS-Cav1 protein vaccine induced CD4^+^ T cells with a T helper-1 (Th1)-biased cytokine profile, making ERD reactions (displaying a Th2 bias) and thus adverse vaccine outcomes unlikely.

Also scientists from Pfizer Inc engineered 400 F-protein variants, which they screened in high-throughput antibody tests. F variant 847 carried a stabilizing engineered disulfide bond and a cavity-filling mutation distinct from DS-Cav1 and a charge neutralization mutation (Che et al. 2023). In cotton rats, F 847 induced 10-fold higher serum Nab titers than DS-Cav1 and complete lung protection was achieved without any evidence of ERD. F 847-bound mabs directed against both epitopes ф and AM14, located at the apex and the lateral side of pre-F, respectively. Pfizer scientists then introduced these substitutions into the F-protein backbones of recently circulating RSV A and B strains. The resulting bivalent vaccine induced comparable Nab titers against both subtypes in vaccinated animals.

## Adult vaccination

### Failure with a post-F vaccine

The superiority of a stabilized pre-F over post-F conformation protein vaccines was demonstrated by using a glucopyranosyl lipid adjuvanted RSV vaccine from MedImmune, containing the RSV F protein in a postfusion conformation (Falloon et al. 2017). The MedImmune vaccine failed to achieve protection against RSV-associated ARI in older subjects. Notably, the vaccine induced a 30-fold increase in F-specific IgG antibody, a 3-fold increase in Nab, and a 10-fold increase in cell-mediated immune response to RSV. Disappointingly, the incidence of RSV-associated ARI was 1.7% and 1.6% in the vaccine and placebo groups, respectively. In a *post hoc* comparison of the immune response induced by the DS-Cav1 pre-F vaccine with that of the MedImmune post-F vaccine, the latter induced significantly lower Nab titers (Chang et al. 2022). B cells preferentially binding the pre-F probe were activated by DS-Cav1, but not by the post-F vaccine.

### Clinical trials with Pfizer’s bivalent pre-F vaccine

Before going into a phase 3 clinical trial with older subjects, Pfizer conducted a challenge study where younger adults received the unadjuvanted bivalent RSV pre-F-protein vaccine (Schmoele-Thoma et al. 2022). One month later the volunteers were challenged intranasally with an RSV A test strain, leading to a symptomatic acute RSV infection in 6% of vaccine and in 42% of the placebo recipients, indicating an 87% vaccine efficacy (VE). Placebo recipients showed a viral load of 10^3^ copies/ml nasal secretion while vaccinated subjects only showed virus titers at the detection limit. Nab titer increased 20-fold in vaccinees and no significant side effects were observed. In a subsequent phase 3 clinical trial, 34 000 adults older than 60 years were enrolled and randomized on the Pfizer vaccine or placebo (Walsh et al. 2023). The trial was conducted between 2021 and 2022 and followed for 7 months thus covering one RSV season in the Northern hemisphere. VE against RSV LRTI displaying two and three clinical symptoms was 67% and 86%, respectively. The higher VE against clinically more important than mild disease (patients with three symptoms showed high percentages of wheezing and shortness of breath while those with two symptoms showed mainly cough) is common in vaccine trials against viral respiratory infections. VE against any acute RSV infection was 62%. No safety concerns were raised except for one case of transient allergic reaction immediately after vaccination and one case of Guillain–Barré syndrome who recovered.

### GSK trials with pre-F vaccine

GlaxoSmithKline (GSK) used the stabilized RSVPreF3 OA (for old age) protein vaccine, approved by the FDA as vaccine (Arexvy) in May 2023 for older adults (Roman et al. 2024). The vaccine was an engineered version of the RSV fusion protein with AS01 adjuvant, a combination of two immunostimulatory compounds. In older subjects, this vaccine increased after two intramuscular injections RSV-specific CD4+ T-cell frequency by 3-fold and neutralizing antibodies to RSV by 6- to 10-fold (Leroux-Roels et al. 2023). In a phase 3 trial, 25 000 older adults from different geographic regions representing various degrees of comorbidities were randomized on a single dose of adjuvanted RSVPreF3 OA or placebo (Papi et al. 2023). After half a year of follow-up in 2021–22, a VE of 83% was observed for subjects from the northern hemisphere. Against severe RSV LRTI VE was 94% and against any RSV ARI still 72% (Feldman et al. 2024). Efficacy against RSV LRTI was >80% in both 60- to 69-year and 70- to 79-year-old groups and in frail subjects. No significant AEs except injection-site pain and fatigue were observed. Subjects with cardiopulmonary or endocrine comorbidities showed comparable Nab increases and the same VE against RSV LRTI as a subject without comorbidities. After a first RSV season, the subjects of the trial were rerandomized and half of the participants received a second dose of the RSVPreF3 OA vaccine while the other half received a placebo (Ison et al. 2024). Revaccination before a second RSV season was well tolerated, but did not significantly increase the VE against severe and nonsevere RSV LRTI, which were 79% and 67%, respectively, in the subsequent season suggesting that vaccination of older adults does not need a yearly boost.

### Moderna mRNA F vaccine

Following the success of mRNA vaccines against SARS-CoV-2, Moderna adapted its rapid and scalable mRNA vaccine platform and developed mRNA-1345, a lipid nanoparticle-encapsulated mRNA-based vaccine encoding the membrane-anchored RSV subtype A F protein, stabilized in pre-F conformation. In a phase 1 trial, mRNA-1345 was used as a single or triple injection in younger adults (Shaw et al. 2024). All subjects had Nab titers at baseline from prior RSV exposure; mRNA-1345 induced 20-fold and 12-fold Nab titer increases against RSV A and RSV B, respectively, and was independent of vaccine dose. Ten-fold increased Nab titers were maintained over 6 months. Repeated mRNA-1345 injections did not boost the Nab titers. Based on these results, mRNA-1345 was used as a single injection in 17 800 older adults, a similar number received a placebo (Wilson et al. 2023). An interim analysis was done after a follow-up of 4 months. No safety concern was raised, and the adverse reactions consisted of transient injection-site pain, headache, and fatigue. VE of mRNA-1345 against RSV LRTI defined by two or three clinical signs was >82%. VE against any RSV ARI was 68%. The protective effect was not attenuated over at least 9 months. Subgroup analysis showed high VE for both sexes, in different age groups, in different geographical regions, and in subjects with frailty and comorbidities. VE was lower against RSV B than against RSV A. As it might be desirable to combine vaccinations against seasonal respiratory viral infections, combinations of mRNA-1345 with inactivated quadrivalent influenza vaccine or with a bivalent SARS-CoV-2 vaccine were explored (Goswami et al. 2024). In a phase 3 trial with adults older than 50 years, the combined vaccines did not increase adverse reactions and elicited mostly noninferior immune responses to RSV compared to individual vaccines.

### Janssen’s adenovirus-vectored F vaccine

Recombinant, replication-incompetent adenovirus serotype 26 (Ad26)-vectored vaccines had proven their efficacy during the COVID-19 pandemic. Therefore, an RSV vaccine was also developed by Janssen Vaccines based on this vector expressing the F protein in its prefusion conformation. The Janssen vaccine contained 10^11^ viral particles of Ad26.RSV.preF complemented with 150 μg of RSV pre-F protein antigen. In the United States, 5800 US adults older than 65 years were randomized on this vaccine or on placebo (Falsey et al. 2023). A quarter of them suffered from chronic cardiac or pulmonary disease. After a single intramuscular injection, VE against RSV LRTI defined by three symptoms was 80%. VE against RSV ARI was 70%. The vaccine increased RSV Nab titers 12-fold and this titer remained 6-fold increased over baseline half a year after vaccination. RSV F-specific T-cell frequency increased 10-fold in vaccines. No safety concerns were reported in alignment with the safety of diverse Ad26-vectored vaccines applied to now >300 000 human subjects. In a follow-up across three RSV seasons, an overall VE against RSV LRTI of 78% was reported (Falsey et al. 2024). Serious AEs over this long follow-up period were balanced between vaccine and placebo recipients.

Information on ongoing developments with live-attenuated RSV vaccine candidates (∆NS2 unpublished, but in phase 3; ∆NS1 in phase 1; mutations in L protein) and RSV vaccines in further virus vectors (parainfluenza virus 5, influenza virus) can be found in Terstappen et al. (2024).

There are only limited real-world data for RSV VE in adults older than 60 years. Among 28 000 RSV-associated hospitalizations of immunocompetent old adults registered in the United States during the 2023–24 RSV season, a test-negative design analysis indicated a VE of 80% for both hospitalization and critical illness (Payne et al. 2024). Among adults with immunocompromising conditions, VE was 73%.

## Maternal vaccination

### Dam vaccination extended

Young infants are particularly susceptible to severe RSV disease and therefore an efficient pediatric RSV vaccine would lighten the burden of RSV disease. However, active immunization in young infants is problematic since maternal RSV antibodies passively transferred to infants might interfere with an injected vaccine in young infants and for concerns about possible ERD in RSV-naïve infants. To circumvent these problems, scientists adapted a practice commonly used in veterinary medicine, namely dam vaccination where antibodies are raised in an animal heavy with a young by active immunization of the dam to protect the newborn young by passively acquired maternal antibodies. This concept was successfully copied in human medicine initially by immunizing pregnant mothers with tetanus toxoid to protect the newborn against neonatal tetanus and later also against pertussis (whooping cough). This approach is particularly attractive because many severe RSV infections occur in the first half year of life when the *in utero* passively acquired maternal antibodies still persisted in the infant’s circulation and because the immune system of the very young infant is still immature.

### Novavax post-F nanoparticle vaccine

Novavax developed an RSV fusion protein F nanoparticle vaccine that contains a recombinant, near-full length F glycoprotein, which was produced in Sf9 insect cells with a recombinant baculovirus (Raghunandan et al. 2014). Purified recombinant RSV F protein oligomers presented a postfusion F-protein conformation. This vaccine raised antibodies directed against antigenic site II of the F protein and prevented RSV replication in the lungs and in the nasal passage of challenged cotton rats. An aluminum-adjuvanted RSV nanoparticle F vaccine induced in pregnant women an ∼10-fold F-protein-specific antibody increase compared to placebo recipients, but only a 2-fold Nab titer increase against RSV-A and no Nab increase to RSV-B (Munoz et al. 2019). The half-life of the transferred antibodies in the children was 40 days. Subsequently, 3000 pregnant women mostly from South Africa and the United States were immunized between 2015 and 2018 with this nanoparticle vaccine (Madhi et al. 2020). Infants from vaccinated mothers showed half as much pneumonia than infants born to placebo receiving mothers (2.2% vs 4.5%), but this parameter was not a predetermined endpoint in the protocol. Endpoints of the clinical protocol were RSV-associated LRTI of various severity for which VEs below 50% were calculated, which did not meet the prespecified success criteria confirming difficulties with vaccines presenting the F protein in a postfusion conformation. With longer follow-up times than 90 days, VE declined even further.

### Pfizer bivalent pre-F vaccine for pregnant mothers

The Pfizer vaccine was tested in 400 pregnant women at two different doses, with or without aluminum hydroxide adjuvant (Simoes et al. 2022). AE consisted mainly of injection-site pain and muscle pain in mothers. RSV Nab titers were at delivery 10- to 17-fold higher in vaccinated than in placebo-receiving mothers, and showed no difference with respect to dose or adjuvant use. An interim VE analysis based on only few RSV cases suggested a tentative VE of 80%. In the subsequent phase 3 MATISSE trial, 7400 pregnant women were enrolled. Within 90 days after birth, 6 of 2800 infants from the vaccine group and 33 of 2800 infants of the placebo group had experienced a severe RSV-associated LRTI indicating a VE of 82%. Within 180 days after birth, VE was 69%. No effect of vaccination was seen for LRTI of any causes. For the women, injection-site pain was the only significant AE. Premature delivery was seen in 0.8% of the vaccine and in 0.6% of the placebo group. Over a 2-year observation period, 0.1% of children of the vaccine and 0.3% of the placebo group had died. The MATISSE trial was conducted between June 2020 and October 2022 during the COVID-19 pandemic (Kampmann et al. 2023). Pfizer’s vaccine was approved in summer 2023 under the trade name Abrysvo by the FDA and by European Medicines Agency (EMA) for use in pregnant women and was recommended by the Centers for Disease Control and Prevention (CDC).

### GSK maternal vaccination trial

In a phase 2 trial, 200 pregnant mothers received one dose of 60- or 120-µg unadjuvanted RSVPreF3 vaccine from GSK, which led to a 10- to 15-fold Nab titer increase over the preimmune titer; no titer increase was seen in women receiving placebo (Bebia et al. 2023). At birth, children from vaccinated mothers showed a 10-fold higher serum Nab titer to RSV than children from placebo mothers. Passive Nab titers showed a half-life of 40 days in infants. As a pregnancy-related AE, a dose-dependent hypertensive disorder of pregnancy was reported (10% vs 5.3% with high and low vaccine dose vs 1.5% in the placebo group). Enrolment stopped with the beginning of the COVID-19 pandemic. A phase 3 clinical trial was stopped for a safety concern when half of the planned 5400 women were enrolled (Dieussaert et al. 2024). A VE of 69% and 66% was calculated against severe and any medically assessed RSV LRTI, respectively. However, vaccinated mothers showed a statistically significant 1.5-fold higher rate of preterm delivery than placebo recipients and a nonsignificant increase in neonatal death (0.4% in vaccine vs 0.2% in the placebo group). The increase in preterm delivery was restricted to a period between April and December 2021 and to women from LMICs, representing half of the enrolled women. An association with infection by the delta variant of SARS-CoV-2 was excluded, and no clear association with the application of further pregnancy vaccines (pertussis, COVID-19, tetanus-diphtheria) was revealed.

## Antivirals

### Need for broad-spectrum antivirals: fluorouridine

So far, standard of care for RSV-infected patients is limited to supportive therapy. The only antiviral to treat an established RSV infection is aerosolized ribavirin, which is approved in the United States for children hospitalized with RSV LRTI. However, its clinical effect is questionable (Tejada et al. 2022). The COVID-19 pandemic has revealed the urgent need for more and novel antivirals. As part of pandemic preparedness, it would be advisable to have a stock of broad-spectrum antivirals that can be quickly used against a number of viral infections, including newly emerging viral threats. In view of the difficulties in developing novel antibiotics against bacterial infections (Brüssow 2024), this might sound like wishful thinking. However, this was exactly the goal of US scientists when tinkering with fluorine substitutions of *N*^4^-hydroxycytidine, the parent compound of the COVID-19 antiviral molnupiravir (Sourimant et al. 2022a). In fact, a 4′-fluorouridine derivative showed in cell culture inhibitory activity not only against RSV, but also against a wide range of viruses covering paramyxoviruses (e.g. measles virus), rhabdoviruses (e.g. rabies virus), and coronaviruses (e.g. SARS-CoV-2), without showing cytotoxicity. *In vitro* experiments with the RSV RNA-dependent RNA polymerase (consisting of recombinant RSV L and P proteins) revealed a stalling of the enzyme when the prodrug 4′-fluorouridine was incorporated instead of Uridine Triphosphate (UTP). In a mouse RSV infection model, oral 4′-fluorouridine reduced lung titers of RSV by 100-fold when given 1 day after virus challenge. Once-daily dosing of 4′-fluorouridine was also sufficient to reduce the titers of SARS-CoV-2 and its variants in a ferret infection model by 10-fold.

### Cytidine nucleoside analogue

Viral load correlates with the clinical manifestations of RSV disease. Therefore, inhibitors of viral replication might represent valuable RSV antivirals. Since replication inhibitors act on intracellular virus, they might also offer a broader therapeutic window than entry inhibitors. ALS-008176 is an orally bioavailable prodrug of a cytidine nucleoside analogue (DeVincenzo et al. 2015). This prodrug is converted intracellularly to a triphosphate nucleoside analogue, which induces chain termination of the RSV replicase. Volunteers experimentally infected with RSV and treated with this prodrug showed a 1000-fold lower viral load and a significant symptom score reduction and decreased nasal secretion compared to placebo recipients. In contrast, fusion inhibitors achieved only a lesser viral load reduction and needed twice as long for RSV to become undetectable than with this nucleoside analogue.

### Presatovir: an RSV entry blocker

Adult healthy volunteer challenge studies have been crucial in the development of neuraminidase inhibitors for the treatment of influenza infections. A number of adult challenge studies have demonstrated activity of several compounds against RSV infection. For example, compound GS-5806 (presatovir), a novel small molecule that inhibits RSV entry by blocking viral–envelope fusion, showed antiviral activity *in vivo*. Adult volunteers were intranasally infected with RSV (DeVincenzo et al. 2014). When the volunteers became positive for RSV replication, they were randomized on oral presatovir or placebo. Presatovir-treated patients showed a notable 4-log titer reduction for nasal RSV, reduced weight of nasal mucus production, and a reduced symptom score. No serious AEs were observed. However, in transplant recipients suffering from acute RSV LRTI, no significant effect of presatovir was observed. The drug was applied 5 days after symptom onset; the lack of an effect might be explained by the fact that at that time point RSV is capable of cell-to-cell spread (Marty et al. 2020). In a parallel clinical trial, transplant patients with an RSV ARI treated with presatovir showed likewise no benefit over placebo treatment (Chemaldy et al. 2020). Researchers sequenced the F gene of the infecting RSV before and after presatovir treatment in 230 patients from four phase 2b trials (Porter et al. 2020). Twenty percent of the presatovir-treated immunosuppressed transplant patients showed presatovir resistance associated substitutions in the F protein compared to 1% in treated nontransplanted RSV patients, confirming concerns of increased mutation rates of RSV in immunocompromised patients.

### Ziresovir fusion inhibitor

Chinese researchers identified in a high throughput screen a benzoazepinequinoline compound with anti-RSV activity. After lead compound identification and further chemical modification they obtained ziresovir (Zheng et al. 2019). Ziresovir demonstrated RSV inhibition in cell culture at nanomolar concentration, had good oral bioavailability, and showed preferential tissue distribution to the lung in experimental animals. Mutants resistant to ziresovir were selected and sequencing identified the same microdomain of the F protein as target as for presatovir. Ziresovir completely inhibited cell-to-cell fusion by RSV in cell culture. In a dose-finding phase 2 clinical trial in infants hospitalized with RSV bronchiolitis, ziresovir showed a greater clinical score and viral load reduction over placebo treatment without raising safety concerns (Huang et al. 2023). In a subsequent phase 3 trial, infants hospitalized with RSV bronchiolitis were treated with ziresovir 4 days after symptom onset for 5 days (Zhao et al. 2024). Ziresovir led to a significant accelerated reduction in clinical bronchiolitis score and the RSV load over placebo starting from the third day of treatment. AEs were rare rash and diarrhea. Nine percent of the infants developed resistance mutations under treatment (<1% under placebo), but no viral rebound was observed.

### Viral factories inhibitor

EDP-938 is a small chemical compound showing in cell culture antiviral activity against RSV at sub-μM concentrations (Rhodin et al. 2021). The compound targets the RSV nucleocapsid N protein and interferes with the buildup of intracellular viral factories visible as inclusion bodies. The compound displayed *in vivo* antiviral activity in a monkey infection model. In a human volunteer infection trial with 115 participants, EDP-938 demonstrated a significant viral load reduction in nasal swabs compared to placebo recipients (Ahmad et al. 2022). Clinical scores and nasal mucus production were reduced in parallel with the viral load reduction. The antiviral effect was observed for a range of oral dosing schemes and no safety issues were raised. A phase 2b clinical trial has been completed, but results have not yet been published.

### Chemical library screens for inhibitors

A screening of 4000 chemical compounds identified cyclopamine, a plant steroidal alkaloid, that inhibited RSV both *in vitro* and in a mouse infection model (Bailly et al. 2016). Cyclopamine inhibits viral RNA transcription. The researchers selected a mutant RSV, which resisted the inhibitory effect of cyclopamine. The mutant displayed a single aa replacement in the RSV transcription factor M2-1. Cyclopamine is an antagonist of the Sonic hedgehog signaling pathway (Shhp) involved in embryonic development, cell differentiation, and tumorigenesis and should thus lead to severe adverse effects. Therefore, the researchers designed an analogue A3E that inhibited RSV but had lost activity for Shhp (Risso-Ballester et al. 2021). They elucidated the mechanism of action of A3E in cell culture experiments. RSV forms in infected cells inclusion bodies containing RSV proteins N, P, L, and M2-1 as well as viral RNA, which represent viral factories mediating viral RNA synthesis. Inclusion bodies are condensates that form by phase separation. A3E blocks RNA replication of RSV by hardening the inclusion body condensate. With bioluminescent RSV as tracer, they demonstrated that A3E inhibits RSV replication in the lung of mice. The development of lung histopathology was suppressed when A3E was given orally up to 1 day after infection.

Screening a 57 000-compound chemical library identified a single hit that blocked the elongation of RSV RNA after initial extension (Cox et al. 2018). Synthetic hit-to-lead exploration approaches yielded compound AVG-233 that demonstrated nanomolar inhibitory activity against RSV in human airway cells (Sourimant et al. 2022b). However, in a mouse RSV infection model, AVG-233 lacked *in vivo* activity because it was rapidly metabolized in lung microsomes. Chemical derivatization led to analogue AVG-388 that displayed a 10-fold RSV viral load reduction in lungs and prevented disease progression to viral pneumonia when given 12 h after infection to mice.

## Conclusions

Substantial progress has been achieved in RSV research over recent years. This is important for several reasons. First, epidemiological research has documented that RSV disease represents a major global disease burden for young infants and older adults. These data are important for public health authorities to decide on the attribution of limited resources for interventions to realize cost-efficient results. Second, the viral world shows a new and threatening dynamic and this applies particularly to respiratory viruses that have caused several pandemics over the last century and continue to produce danger signals of newly emerging viral threats.

Prophylaxis of newborns with nirsevimab is now routine in several industrialized countries ahead of annual RSV epidemics. The advances in passive and active immunization approaches against RSV thus represent important interventions not only for alleviating RSV morbidity and mortality, but to highlight countermeasures against viral respiratory infections in general. The endemicity of RSV infection was a motivational motor for recent technological progress. Case numbers are high and occur in regular yearly epidemics, which facilitate the planning of clinical trials. In addition, regular epidemics of public health importance assure markets for pharmaceutical products and thus provide a financial incentive to invest in large clinical trials on active and passive immunity approaches against RSV expecting a return on investments.

Important technological developments have been realized when developing active and passive immunity against RSV. Particularly fruitful has been the synergy between structural biology and protein engineering of the RSV F protein, which allowed to stabilize the RSV prefusion protein conformation. Structural biology and protein engineering are both platform technologies that can be easily transferred to pathogens from other viral infections including newly emerging respiratory viruses. Other platform technologies such as adenovirus-vectored vaccines or mRNA vaccines against RSV infections are under development. This would not only extend the knowledge acquired with these new vaccine platforms from SARS-CoV-2 to RSV but might help to develop them also relatively quickly against future pandemic threat viruses. However, mRNA vaccine development against RSV in children has recently seen a setback when US regulators paused clinical trials when the industrial sponsor Moderna reported that five infants receiving the mRNA vaccine developed severe disease after experiencing a natural infection, versus one in the placebo group (doi: 10.1126/science.zz245f8). If confirmed, it will be a new hurdle to the development of active immunization against RSV in infants. Therefore a valuable aspect of recent RSV research is efforts to explore novel antivirals based on new mechanisms of action, which hopefully will stimulate antiviral research against other RNA viruses and will also reveal how RNA viruses can acquire mutations that allow them to escape from control by novel antivirals.

## References

[bib1] Abu-Raya B, Viñeta Paramo M, Reicherz F et al. Why has the epidemiology of RSV changed during the COVID-19 pandemic?. Eclinicalmedicine. 2023;61:102089. 10.1016/j.eclinm.2023.102089 37483545 PMC10359735

[bib2] Achten NB, Wu P, Bont L et al. Interference between respiratory syncytial virus and human rhinovirus infection in infancy. J Infect Dis. 2017;215:1102–6. 10.1093/infdis/jix031 28368456 PMC5426371

[bib3] Acosta PL, Caballero MT, Polack FP. Brief history and characterization of enhanced respiratory syncytial virus disease. Clin Vaccine Immunol. 2016;23:189–95. 10.1128/CVI.00609-15 PMC4783420 26677198

[bib4] Adams G, Moreno GK, Petros BA et al. Viral lineages in the 2022 RSV surge in the United States. N Engl J Med. 2023;388:1335–7. 10.1056/NEJMc2216153 36812457 PMC10081154

[bib5] Ahmad A, Eze K, Noulin N et al. EDP-938, a respiratory syncytial virus inhibitor, in a human virus challenge. N Engl J Med. 2022;386:655–66. 10.1056/NEJMoa2108903 35172056

[bib6] Allen DM, Reyne MI, Allingham P et al. Genomic analysis and surveillance of respiratory syncytial virus (RSV) using wastewater-based epidemiology (WBE). J Infect Dis. 2024;230:e895. 10.1093/infdis/jiae205 38636496 PMC11481326

[bib7] Ares-Gómez S, Mallah N, Santiago-Pérez MI et al. Effectiveness and impact of universal prophylaxis with nirsevimab in infants against hospitalisation for respiratory syncytial virus in Galicia, Spain: initial results of a population-based longitudinal study. Lancet Infect Dis. 2024;24:817–28. 10.1016/S1473-3099(24)00215-9 38701823

[bib8] Assad Z, Romain AS, Aupiais C et al. Nirsevimab and hospitalization for RSV bronchiolitis. N Engl J Med. 2024;391:144–54. 10.1056/NEJMoa2314885 38986058

[bib9] Bailly B, Richard CA, Sharma G et al. Targeting human respiratory syncytial virus transcription anti-termination factor M2-1 to inhibit in vivo viral replication. Sci Rep. 2016;6:25806. 10.1038/srep25806 27194388 PMC4872165

[bib10] Bardsley M, Morbey RA, Hughes HE et al. Epidemiology of respiratory syncytial virus in children younger than 5 years in England during the COVID-19 pandemic, measured by laboratory, clinical, and syndromic surveillance: a retrospective observational study. Lancet Infect Dis. 2023;23:56–66. 10.1016/S1473-3099(22)00525-4 36063828 PMC9762748

[bib11] Bebia Z, Reyes O, Jeanfreau R et al. Safety and immunogenicity of an investigational respiratory syncytial virus vaccine (RSVPreF3) in mothers and their infants: a phase 2 randomized trial. J Infect Dis. 2023;228:299–310. 10.1093/infdis/jiad024 36722147 PMC10420396

[bib12] Billard MN, Bont LJ. Quantifying the RSV immunity debt following COVID-19: a public health matter. Lancet Infect Dis. 2023;23:3–5. 10.1016/S1473-3099(22)00544-8 36063827 PMC9439700

[bib13] Brüssow H, Werchau H, Sidoti J et al. Age-related prevalence of serum antibody to respiratory syncytial virus in Ecuadorian and German children. J Infect Dis. 1991;163:679–80. 10.1093/infdis/163.3.679a 1995747

[bib14] Brüssow H . The antibiotic resistance crisis and the development of new antibiotics. Microb Biotechnol. 2024;17:e14510. 10.1111/1751-7915.14510 38970161 PMC11226406

[bib15] Buchholz UJ, Anderson LJ, Collins PL et al. Respiratory syncytical virus and metapneumovirus. In: Howley PM, Knipe DM (eds), Field's Virology. Vol 3: RNA Viruses. Philadelphia: Wolters Kluwer Publisher, 2023, 267–317.

[bib16] Caserta MT, O'Leary ST, Munoz FM et al. Palivizumab prophylaxis in infants and young children at increased risk of hospitalization for respiratory syncytial virus infection. Pediatrics. 2023;152:e2023061803. 10.1542/peds.2023-061803 37357729

[bib17] Chang LA, Phung E, Crank MC et al. A prefusion-stabilized RSV F subunit vaccine elicits B cell responses with greater breadth and potency than a postfusion F vaccine. Sci Transl Med. 2022;14:eade0424. 10.1126/scitranslmed.ade0424 36542692 PMC11345946

[bib18] Che Y, Gribenko AV, Song X et al. Rational design of a highly immunogenic prefusion-stabilized F glycoprotein antigen for a respiratory syncytial virus vaccine. Sci Transl Med. 2023;15:eade6422. 10.1126/scitranslmed.ade6422 37023209

[bib19] Chemaly RF, Dadwal SS, Bergeron A et al. A phase 2, randomized, double-blind, placebo-controlled trial of presatovir for the treatment of respiratory syncytial virus upper respiratory tract infection in hematopoietic-cell transplant recipients. Clin Infect Dis. 2020;71:2777–86. 10.1093/cid/ciz1166 31793991 PMC7108134

[bib20] Colosia A, Costello J, McQuarrie K et al. Systematic literature review of the signs and symptoms of respiratory syncytial virus. Influenza Resp Viruses. 2023;17:e13100. 10.1111/irv.13100 PMC9899685 36824394

[bib21] Cong B, Koç U, Bandeira T et al. Changes in the global hospitalisation burden of respiratory syncytial virus in young children during the COVID-19 pandemic: a systematic analysis. Lancet Infect Dis. 2024;24:361–74. 10.1016/S1473-3099(23)00630-8 38141633 PMC11290460

[bib22] Cox RM, Toots M, Yoon JJ et al. Development of an allosteric inhibitor class blocking RNA elongation by the respiratory syncytial virus polymerase complex. J Biol Chem. 2018;293:16761–77. 10.1074/jbc.RA118.004862 30206124 PMC6204889

[bib23] Crank MC, Ruckwardt TJ, Chen M et al. A proof of concept for structure-based vaccine design targeting RSV in humans. Science. 2019;365:505–9. 10.1126/science.aav9033 31371616

[bib24] Cunningham S, Piedra PA, Martinon-Torres F et al. Nebulised ALX-0171 for respiratory syncytial virus lower respiratory tract infection in hospitalised children: a double-blind, randomised, placebo-controlled, phase 2b trial. Lancet Respir Med. 2021;9:21–32. 10.1016/S2213-2600(20)30320-9 33002427

[bib25] Dallagiacoma G, Arthur Rhedin S, Odone A et al. A comparative analysis of non-pharmaceutical interventions for preventing the respiratory syncytial virus in 30 European countries. Acta Paediatr. 2024;113:1388–95. 10.1111/apa.17199 38453683

[bib26] Delgado MF, Coviello S, Monsalvo AC et al. Lack of antibody affinity maturation due to poor toll-like receptor stimulation leads to enhanced respiratory syncytial virus disease. Nat Med. 2009;15:34–41. 10.1038/nm.1894 19079256 PMC2987729

[bib27] den Hartog G, van Kasteren PB, Schepp RM et al. Decline of RSV-specific antibodies during the COVID-19 pandemic. Lancet Infect Dis. 2023;23:23–25. 10.1016/S1473-3099(22)00763-0 36463892 PMC9714975

[bib28] DeVincenzo JP, McClure MW, Symons JA et al. Activity of oral ALS-008176 in a respiratory syncytial virus challenge study. N Engl J Med. 2015;373:2048–58. 10.1056/NEJMoa1413275 26580997

[bib29] DeVincenzo JP, Whitley RJ, Mackman RL et al. Oral GS-5806 activity in a respiratory syncytial virus challenge study. N Engl J Med. 2014;371:711–22. 10.1056/NEJMoa1401184 25140957

[bib30] Dieussaert I, Hyung Kim J, Luik S et al. RSV prefusion F protein-based maternal vaccine—preterm birth and other outcomes. N Engl J Med. 2024;390:1009–21. 10.1056/NEJMoa2305478 38477988

[bib31] Domachowske J, Madhi SA, Simões EAF et al. Safety of nirsevimab for RSV in infants with heart or lung disease or prematurity. N Engl J Med. 2022;386:892–4. 10.1056/NEJMc2112186 35235733

[bib32] Drysdale SB, Cathie K, Flamein F et al. Nirsevimab for prevention of hospitalizations due to RSV in infants. N Engl J Med. 2023;389:2425–35. 10.1056/NEJMoa2309189 38157500

[bib34] Falloon J, Yu J, Esser MT et al. An adjuvanted, postfusion F protein-based vaccine did not prevent respiratory syncytial virus illness in older adults. J Infect Dis. 2017;216:1362–70. 10.1093/infdis/jix503 29029260 PMC5853767

[bib35] Falsey AR, Hennessey PA, Formica MA et al. Respiratory syncytial virus infection in elderly and high-risk adults. N Engl J Med. 2005;352:1749–59. 10.1056/NEJMoa043951 15858184

[bib36] Falsey AR, Hosman T, Bastian AR et al. Long-term efficacy and immunogenicity of Ad26.RSV.PreF-RSV preF protein vaccine (CYPRESS): a randomised, double-blind, placebo-controlled, phase 2b study. Lancet Infect Dis. 2024;24:1015–24. 10.1016/S1473-3099(24)00226-3 38801826

[bib38] Falsey AR, Williams K, Gymnopoulou E et al. Efficacy and safety of an Ad26.RSV.PreF-RSV preF protein vaccine in older adults. N Engl J Med. 2023;388:609–20. 10.1056/NEJMoa2207566 36791161

[bib39] Feikin DR, Karron RA, Saha SK et al. The full value of immunisation against respiratory syncytial virus for infants younger than 1 year: effects beyond prevention of acute respiratory illness. Lancet Infect Dis. 2024;24:e318–27. 10.1016/S1473-3099(23)00568-6 38000374

[bib40] Feldman RG, Antonelli-Incalzi R, Steenackers K et al. Respiratory syncytial virus prefusion F protein vaccine is efficacious in older adults with underlying medical conditions. Clin Infect Dis. 2024;78:202–9. 10.1093/cid/ciad471 37698366 PMC10810713

[bib41] Foley DA, Yeoh DK, Minney-Smith CA et al. The interseasonal resurgence of respiratory syncytial virus in Australian children following the reduction of coronavirus disease 2019-related public health measures. Clin Infect Dis. 2021;73:e2829–30. 10.1093/cid/ciaa1906 33594407 PMC7929151

[bib42] Fourati S, Reslan A, Bourret J et al. Genotypic and phenotypic characterisation of respiratory syncytial virus after nirsevimab breakthrough infections: a large, multicentre, observational, real-world study. Lancet Infect Dis. 10.1016/S1473-3099(24)00570-X, 14 October2024,; preprint: not peer reviewed. 39419046

[bib43] Franz A, Adams O, Willems R et al. Correlation of viral load of respiratory pathogens and co-infections with disease severity in children hospitalized for lower respiratory tract infection. J Clin Virol. 2010;48:239–45. 10.1016/j.jcv.2010.05.007 20646956 PMC7185496

[bib44] GBD 2019 LRI Collaborators . Age-sex differences in the global burden of lower respiratory infections and risk factors, 1990-2019: results from the Global Burden of Disease Study 2019. Lancet Infect Dis. 2022;22:1626–47. 10.1016/S1473-3099(22)00510-2 35964613 PMC9605880

[bib45] GBD 2016 Lower Respiratory Infections Collaborators. Estimates of the global, regional, and national morbidity, mortality, and aetiologies of lower respiratory infections in 195 countries, 1990-2016: a systematic analysis for the Global Burden of Disease Study 2016. Lancet Infect Dis. 2018;18:1191–210. 10.1016/S1473-3099(18)30310-4 30243584 PMC6202443

[bib46] Gonzales T, Bergamasco A, Cristarella T et al. Effectiveness and safety of palivizumab for the prevention of serious lower respiratory tract infection caused by respiratory syncytial virus: a systematic review. Am J Perinatol. 2024;41:e1107–15. 10.1055/a-1990-2633 36452969 PMC11108679

[bib47] Goswami J, Cardona JF, Hsu DC et al. Safety and immunogenicity of mRNA-1345 RSV vaccine coadministered with an influenza or COVID-19 vaccine in adults aged 50 years or older: an observer-blinded, placebo-controlled, randomised, phase 3 trial. Lancet Infect Dis. 10.1016/S1473-3099(24)00589-9, 25 November2024 preprint: not peer reviewed. 39608389

[bib48] Griffin MP, Yuan Y, Takas T et al. Single-dose nirsevimab for prevention of RSV in preterm infants. N Engl J Med. 2020;383:415–25. 10.1056/NEJMoa1913556 32726528

[bib49] Guiñazú G, Dvorkin J, Mahmud S et al. Evaluation of the potential impact and cost-effectiveness of respiratory syncytial virus (RSV) prevention strategies for infants in Argentina. Vaccine. 2024;42:126234. 10.1016/j.vaccine.2024.126234 39154512 PMC11413482

[bib50] Haddadin Z, Beveridge S, Fernandez K et al. Respiratory syncytial virus disease severity in young children. Clin Infect Dis. 2021;73:e4384–91. 10.1093/cid/ciaa1612 33095882 PMC8826377

[bib51] Hall CB, Weinberg GA, Iwane MK et al. The burden of respiratory syncytial virus infection in young children. N Engl J Med. 2009;360:588–98. 10.1056/NEJMoa0804877 19196675 PMC4829966

[bib52] Hammitt LL, Dagan R, Yuan Y et al. Nirsevimab for prevention of RSV in healthy late-preterm and term infants. N Engl J Med. 2022;386:837–46. 10.1056/NEJMoa2110275 35235726

[bib53] Haney J, Vijayakrishnan S, Streetley J et al. Coinfection by influenza A virus and respiratory syncytial virus produces hybrid virus particles. Nat Microbiol. 2022;7:1879–90. 10.1038/s41564-022-01242-5 36280786

[bib54] Hansen CL, Chaves SS, Demont C et al. Mortality associated with influenza and respiratory syncytial virus in the US, 1999-2018. JAMA Netw Open. 2022;5:e220527. 10.1001/jamanetworkopen.2022.0527 35226079 PMC8886548

[bib55] Huang LM, Schibler A, Huang YC et al. Safety and efficacy of AK0529 in respiratory syncytial virus-infected infant patients: a phase 2 proof-of-concept trial. Influenza Resp Viruses. 2023;17:e13176. 10.1111/irv.13176 PMC10368966 37502622

[bib56] Ison MG, Papi A, Athan E et al. Efficacy and safety of respiratory syncytial virus (RSV) prefusion F protein vaccine (RSVPreF3 OA) in older adults over 2 RSV seasons. Clin Infect Dis. 2024;78:1732–44. 10.1093/cid/ciae010 38253338 PMC11175669

[bib57] Kampmann B, Madhi SA, Munjal I et al. Bivalent prefusion F vaccine in pregnancy to prevent RSV illness in infants. N Engl J Med. 2023;388:1451–64. 10.1056/NEJMoa2216480 37018474

[bib58] Kapikian AZ, Mitchell RH, Chanock RM et al. An epidemiologic study of altered clinical reactivity to respiratory syncytial (RS) virus infection in children previously vaccinated with an inactivated RS virus vaccine. Am J Epidemiol. 1969;89:405–21. 10.1093/oxfordjournals.aje.a120954 4305197

[bib59] Kim HW, Canchola JG, Brandt CD et al. Respiratory syncytial virus disease in infants despite prior administration of antigenic inactivated vaccine. Am J Epidemiol. 1969;89:422–34. 10.1093/oxfordjournals.aje.a120955 4305198

[bib60] Koltai M, Moyes J, Nyawanda B et al. Estimating the cost-effectiveness of maternal vaccination and monoclonal antibodies for respiratory syncytial virus in Kenya and South Africa. BMC Med. 2023;21:120. 10.1186/s12916-023-02806-w 37004062 PMC10064962

[bib61] Krarup A, Truan D, Furmanova-Hollenstein P et al. A highly stable prefusion RSV F vaccine derived from structural analysis of the fusion mechanism. Nat Commun. 2015;6:8143. 10.1038/ncomms9143 PMC4569726 26333350

[bib62] Lee N, Lui GC, Wong KT et al. High morbidity and mortality in adults hospitalized for respiratory syncytial virus infections. Clin Infect Dis. 2013;57:1069–77. 10.1093/cid/cit471 23876395

[bib63] Leroux-Roels I, Davis MG, Steenackers K et al. Safety and immunogenicity of a respiratory syncytial virus prefusion F (RSVPreF3) candidate vaccine in older adults: phase 1/2 randomized clinical trial. J Infect Dis. 2023;227:761–72. 10.1093/infdis/jiac327 35904987 PMC10044090

[bib64] Li X, Willem L, Antillon M et al. Health and economic burden of respiratory syncytial virus (RSV) disease and the cost-effectiveness of potential interventions against RSV among children under 5 years in 72 Gavi-eligible countries. BMC Med. 2020;18:82. 10.1186/s12916-020-01537-6 32248817 PMC7132892

[bib65] Li Y, Wang X, Blau DM et al. Global, regional, and national disease burden estimates of acute lower respiratory infections due to respiratory syncytial virus in children younger than 5 years in 2019: a systematic analysis. Lancet. 2022;399:2047–64. 10.1016/S0140-6736(22)00478-0 35598608 PMC7613574

[bib66] Luo Z, Fu Z, Liu E et al. Nebulized hypertonic saline treatment in hospitalized children with moderate to severe viral bronchiolitis. Clin Microbiol Infect. 2011;17:1829–33. 10.1111/j.1469-0691.2010.03304.x 20636429

[bib67] Madhi SA, Polack FP, Piedra PA et al. Respiratory syncytial virus vaccination during pregnancy and effects in infants. N Engl J Med. 2020;383:426–39. 10.1056/NEJMoa1908380 32726529 PMC7299433

[bib68] Mahmud S, Baral R, Sanderson C et al. Cost-effectiveness of pharmaceutical strategies to prevent respiratory syncytial virus disease in young children: a decision-support model for use in low-income and middle-income countries. BMC Med. 2023;21:138. 10.1186/s12916-023-02827-5 37038127 PMC10088159

[bib69] Marty FM, Chemaly RF, Mullane KM et al. A phase 2b, randomized, double-blind, placebo-controlled multicenter study evaluating antiviral effects, pharmacokinetics, safety, and tolerability of presatovir in hematopoietic cell transplant recipients with respiratory syncytial virus infection of the lower respiratory tract. Clin Infect Dis. 2020;71:2787–95. 10.1093/cid/ciz1167 31915807 PMC7108198

[bib70] Mazur NI, Caballero MT, Nunes MC. Severe respiratory syncytial virus infection in children: burden, management, and emerging therapies. Lancet. 2024;404:1143–56. 10.1016/S0140-6736(24)01716-1 39265587

[bib71] McLaughlin JM, Khan F, Begier E et al. Rates of medically attended RSV among US adults: a systematic review and meta-analysis. Open Forum Infect Dis. 2022;9:ofac300. 10.1093/ofid/ofac300 35873302 PMC9301578

[bib72] McLellan JS, Chen M, Joyce MG et al. Structure-based design of a fusion glycoprotein vaccine for respiratory syncytial virus. Science. 2013a;342:592–8. 10.1126/science.1243283 24179220 PMC4461862

[bib73] McLellan JS, Chen M, Leung S et al. Structure of RSV fusion glycoprotein trimer bound to a prefusion-specific neutralizing antibody. Science. 2013b;340:1113–7. 10.1126/science.1234914 23618766 PMC4459498

[bib74] Meissner HC . Viral bronchiolitis in children. N Engl J Med. 2016;374:62–72. 10.1056/NEJMra1413456 26735994

[bib75] Messacar K, Baker RE, Park SW et al. Preparing for uncertainty: endemic paediatric viral illnesses after COVID-19 pandemic disruption. Lancet. 2022;400:1663–5. 10.1016/S0140-6736(22)01277-6 35843260 PMC9282759

[bib76] Moghaddam A, Olszewska W, Wang B et al. A potential molecular mechanism for hypersensitivity caused by formalin-inactivated vaccines. Nat Med. 2006;12:905–7. 10.1038/nm1456 16862151

[bib77] Muller WJ, Madhi SA, Seoane Nuñez B et al. Nirsevimab for prevention of RSV in term and late-preterm infants. N Engl J Med. 2023;388:1533–4. 10.1056/NEJMc2214773 37018470

[bib78] Muňoz FM, Swamy GK, Hickman SP et al. Safety and immunogenicity of a respiratory syncytial virus fusion (F) protein nanoparticle vaccine in healthy third-trimester pregnant women and their infants. J Infect Dis. 2019;220:1802–15. 10.1093/infdis/jiz390 31402384

[bib79] Nickbakhsh S, Thorburn F, von Wissmann B et al. Extensive multiplex PCR diagnostics reveal new insights into the epidemiology of viral respiratory infections. Epidemiol Infect. 2016;144:2064–76. 10.1017/S0950268816000339 26931455 PMC7113017

[bib80] Nygaard U, Hartling UB, Nielsen J et al. Hospital admissions and need for mechanical ventilation in children with respiratory syncytial virus before and during the COVID-19 pandemic: a Danish nationwide cohort study. Lancet Child & Adolesc Health. 2023;7:171–9. 10.1016/S2352-4642(22)00371-6 PMC9940917 36634692

[bib81] Olson MR, Varga SM. CD8 T cells inhibit respiratory syncytial virus (RSV) vaccine-enhanced disease. J Immunol. 2007;179:5415–24. 10.4049/jimmunol.179.8.5415 17911628

[bib82] Orsi A, Scarpaleggia M, Baldo V et al. First real-world data on universal respiratory syncytial virus prophylaxis with nirsevimab in infants. J Prev Med Hyg. 2024;65:E172–87. 10.15167/2421-4248/jpmh2024.65.2.3329 39430977 PMC11487721

[bib83] Papi A, Ison MG, Langley JM et al. Respiratory syncytial virus prefusion F protein vaccine in older adults. N Engl J Med. 2023;388:595–608. 10.1056/NEJMoa2209604 36791160

[bib84] Payne AB, Watts JA, Mitchell PK et al. Respiratory syncytial virus (RSV) vaccine effectiveness against RSV-associated hospitalisations and emergency department encounters among adults aged 60 years and older in the USA, October, 2023, to March, 2024: a test-negative design analysis. Lancet. 2024;404:1547–59. 10.1016/S0140-6736(24)01738-0 39426837 PMC13286031

[bib85] Pecenka C, Sparrow E, Feikin DR et al. Respiratory syncytial virus vaccination and immunoprophylaxis: realising the potential for protection of young children. Lancet. 2024;404:1157–70. 10.1016/S0140-6736(24)01699-4 39265588

[bib86] Piret J, Boivin G. Viral interference between respiratory viruses. Emerg Infect Dis. 2022;28:273–81. 10.3201/eid2802.211727 35075991 PMC8798701

[bib87] Polack FP, Alvarez-Paggi D, Libster R et al. Fatal enhanced respiratory syncytial virus disease in toddlers. Sci Transl Med. 2021;13:eabj7843. 10.1126/scitranslmed.abj7843 34669442 PMC10712289

[bib88] Polack FP, Teng MN, Collins PL et al. A role for immune complexes in enhanced respiratory syncytial virus disease. J Exp Med. 2002;196:859–65. 10.1084/jem.20020781 12235218 PMC2194058

[bib89] Porter DP, Guo Y, Perry J et al. Assessment of drug resistance during phase 2b clinical trials of presatovir in adults naturally infected with respiratory syncytial virus. Antimicrob Agents Chemother. 2020;64:e02312–19. 10.1128/AAC.02312-19 32071058 PMC7449164

[bib90] Raghunandan R, Lu H, Zhou B et al. An insect cell derived respiratory syncytial virus (RSV) F nanoparticle vaccine induces antigenic site II antibodies and protects against RSV challenge in cotton rats by active and passive immunization. Vaccine. 2014;32:6485–92. 10.1016/j.vaccine.2014.09.030 25269094 PMC7172787

[bib91] Reicherz F, Xu RY, Abu-Raya B et al. Waning immunity against respiratory syncytial virus during the coronavirus disease 2019 pandemic. J Infect Dis. 2022;226:2064–8. 35524952 10.1093/infdis/jiac192 PMC9129162

[bib92] Rhodin MHJ, McAllister NV, Castillo J et al. EDP-938, a novel nucleoprotein inhibitor of respiratory syncytial virus, demonstrates potent antiviral activities in vitro and in a non-human primate model. PLoS Pathog. 2021;17:e1009428. 10.1371/journal.ppat.1009428 33720995 PMC7993833

[bib93] Risso-Ballester J, Galloux M, Cao J et al. A condensate-hardening drug blocks RSV replication in vivo. Nature. 2021;595:596–9. 10.1038/s41586-021-03703-z 34234347

[bib94] Roman F, Burny W, Ceregido MA et al. Adjuvant system AS01: from mode of action to effective vaccines. Expert Rev Vaccines. 2024;23:715–29. 10.1080/14760584.2024.2382725 39042099

[bib95] Savic M, Penders Y, Shi T et al. Respiratory syncytial virus disease burden in adults aged 60 years and older in high-income countries: a systematic literature review and meta-analysis. Influenza Resp Viruses. 2023;17:e13031. 10.1111/irv.13031 PMC9835463 36369772

[bib96] Schmoele-Thoma B, Zareba AM, Jiang Q et al. Vaccine efficacy in adults in a respiratory syncytial virus challenge study. N Engl J Med. 2022;386:2377–86. 10.1056/NEJMoa2116154. 35731653

[bib97] Shaw CA, Mithani R, Kapoor A et al. Safety, tolerability and immunogenicity of a mRNA-based RSV vaccine in healthy young adults in a phase 1 clinical trial. J Infect Dis. 2024;230:e637. 10.1093/infdis/jiae035 38298125 PMC11420805

[bib98] Shi T, McAllister DA, O'Brien KL et al. Global, regional, and national disease burden estimates of acute lower respiratory infections due to respiratory syncytial virus in young children in 2015: a systematic review and modelling study. Lancet. 2017;390:946–58. 10.1016/S0140-6736(17)30938-8 28689664 PMC5592248

[bib99] Simões EAF, Center KJ, Tita ATN et al. Prefusion F protein-based respiratory syncytial virus immunization in pregnancy. N Engl J Med. 2022;386:1615–26. 10.1056/NEJMoa2106062 35476650

[bib100] Simões EAF, Madhi SA, Muller WJ et al. Efficacy of nirsevimab against respiratory syncytial virus lower respiratory tract infections in preterm and term infants, and pharmacokinetic extrapolation to infants with congenital heart disease and chronic lung disease: a pooled analysis of randomised controlled trials. Lancet Child Adolesc Health. 2023;7:180–9. 10.1016/S2352-4642(22)00321-2 36634694 PMC9940918

[bib101] Sourimant J, Lieber CM, Aggarwal M et al. 4'-Fluorouridine is an oral antiviral that blocks respiratory syncytial virus and SARS-CoV-2 replication. Science. 2022a;375:161–7. 10.1126/science.abj5508 34855509 PMC9206510

[bib102] Sourimant J, Lieber CM, Yoon JJ et al. Orally efficacious lead of the AVG inhibitor series targeting a dynamic interface in the respiratory syncytial virus polymerase. Sci Adv. 2022b;8:eabo2236. 10.1126/sciadv.abo2236 35749502 PMC9232112

[bib103] Tejada S, Martinez-Reviejo R, Karakoc HN et al. Ribavirin for treatment of subjects with respiratory syncytial virus-related infection: a systematic review and meta-analysis. Adv Ther. 2022;39:4037–51. 10.1007/s12325-022-02256-5 35876973

[bib104] Terstappen J, Hak SF, Bhan A et al. The respiratory syncytial virus vaccine and monoclonal antibody landscape: the road to global access. Lancet Infect Dis. 2024;24:e747–61. 10.1016/S1473-3099(24)00455-9 39326422 PMC12311909

[bib105] Toepfer AP, Amarin JZ, Spieker AJ et al. Seasonality, clinical characteristics, and outcomes of respiratory syncytial virus disease by subtype among children aged <5 years: new Vaccine Surveillance Network, United States, 2016-2020. Clin Infect Dis. 2024;78:1352–9. 10.1093/cid/ciae085 38366649 PMC11093674

[bib106] Varga SM . Fixing a failed vaccine. Nat Med. 2009;15:21–22. 10.1038/nm0109-21 19129777

[bib107] Walsh EE, Pérez Marc G, Zareba AM et al. Efficacy and safety of a bivalent RSV prefusion F vaccine in older adults. N Engl J Med. 2023;388:1465–77. 10.1056/NEJMoa2213836 37018468

[bib108] Wang X, Li Y, Shi T et al. Global disease burden of and risk factors for acute lower respiratory infections caused by respiratory syncytial virus in preterm infants and young children in 2019: a systematic review and meta-analysis of aggregated and individual participant data. Lancet. 2024;403:1241–53. 10.1016/S0140-6736(24)00138-7 38367641

[bib109] Wildenbeest JG, Billard MN, Zuurbier RP et al. The burden of respiratory syncytial virus in healthy term-born infants in Europe: a prospective birth cohort study. Lancet Respir Med. 2023;11:341–53. 36372082 10.1016/S2213-2600(22)00414-3 PMC9764871

[bib110] Wilkins D, Langedijk AC, Lebbink RJ et al. Nirsevimab binding-site conservation in respiratory syncytial virus fusion glycoprotein worldwide between 1956 and 2021: an analysis of observational study sequencing data. Lancet Infect Dis. 2023;23:856–66. 10.1016/S1473-3099(23)00062-2 36940703

[bib111] Wilson E, Goswami J, Baqui AH et al. Efficacy and safety of an mRNA-based RSV PreF vaccine in older adults. N Engl J Med. 2023;389:2233–44. 10.1056/NEJMoa2307079 38091530

[bib112] Winthrop ZA, Perez JM, Staffa SJ et al. Pediatric respiratory syncytial virus hospitalizations and respiratory support after the COVID-19 pandemic. JAMA Netw Open. 2024;7:e2416852. 10.1001/jamanetworkopen.2024.16852 38869896 PMC11177168

[bib113] Ye RZ, Que TC, Xia LY et al. Natural infection of pangolins with human respiratory syncytial viruses. Curr Biol. 2022;32:R307–8. 10.1016/j.cub.2022.02.057 35413253

[bib114] Zar HJ, Cacho F, Kootbodien T et al. Early-life respiratory syncytial virus disease and long-term respiratory health. Lancet Respir Med. 2024;12:810–21. 10.1016/S2213-2600(24)00246-7 39265601

[bib115] Zar HJ, Nduru P, Stadler JAM et al. Early-life respiratory syncytial virus lower respiratory tract infection in a South African birth cohort: epidemiology and effect on lung health. Lancet Glob Health. 2020;8:e1316–25. 10.1016/S2214-109X(20)30251-5 32971054 PMC7511798

[bib116] Zhao S, Shang Y, Yin Y et al. Ziresovir in hospitalized infants with respiratory syncytial virus infection. N Engl J Med. 2024;391:1096–107. 10.1056/NEJMoa2313551 39321361

[bib117] Zheng X, Gao L, Wang L et al. Discovery of ziresovir as a potent, selective, and orally bioavailable respiratory syncytial virus fusion protein inhibitor. J Med Chem. 2019;62:6003–14. 10.1021/acs.jmedchem.9b00654 31194544

[bib118] Zheng Z, Pitzer VE, Warren JL et al. Community factors associated with local epidemic timing of respiratory syncytial virus: a spatiotemporal modeling study. Sci Adv. 2021;7:eabd6421. 10.1126/sciadv.abd6421 34162556 PMC8221622

[bib119] Zhu Q, McLellan JS, Kallewaard NL et al. A highly potent extended half-life antibody as a potential RSV vaccine surrogate for all infants. Sci Transl Med. 2017;9:eaaj1928. 10.1126/scitranslmed.aaj1928 28469033

